# Projections of care for older people with dementia in England: 2015 to 2040

**DOI:** 10.1093/ageing/afz154

**Published:** 2019-12-06

**Authors:** Raphael Wittenberg, Bo Hu, Carol Jagger, Andrew Kingston, Martin Knapp, Adelina Comas-Herrera, Derek King, Amritpal Rehill, Sube Banerjee

**Affiliations:** 1 Care Policy and Evaluation Centre, London School of Economics and Political Science, London, UK; 2 Population Health Sciences Institute, Newcastle University, Newcastle, UK; 3 Faculty of Health, University of Plymouth, Plymouth, UK

**Keywords:** *dementia*, *projections*, *costs*, *services*

## Abstract

**Background:**

The number of older people with dementia and the cost of caring for them, already substantial, are expected to rise due to population ageing.

**Objective:**

This study makes projections of the number of older people with dementia receiving unpaid care or using care services and associated costs in England.

**Methods:**

The study drew on up-to-date information for England from multiple sources including data from the CFASII study, output from the PACSim dynamic microsimulation model, Office for National Statistics population projections and data from the MODEM cohort study. A simulation model was built to make the projections.

**Results:**

We project that the number of older people with dementia will more than double in the next 25 years. The number receiving unpaid or formal care is projected to rise by 124%, from 530,000 in 2015 to 1,183,000 in 2040. Total cost of dementia is projected to increase from £23.0 billion in 2015 to £80.1 billion in 2040, and average cost is projected to increase from £35,100 per person per year in 2015 to £58,900 per person per year in 2040. Total and average costs of social care are projected to increase much faster than those of healthcare and unpaid care.

**Conclusion:**

The numbers of people with dementia and associated costs of care will rise substantially in the coming decades, unless new treatments enable the progression of the condition to be prevented or slowed. Care and support for people with dementia and their family carers will need to be increased.

## Introduction

Recent decades have seen a large increase in life expectancy due in part to a substantial decline in mortality rates in old age. With increased longevity comes an increased risk of experiencing disorders, which increase in prevalence with increasing age, such as dementia. The projected rise in number of people aged 75 and over and 85 and over has fuelled concern about how best to plan for and finance the care of rising numbers of people with dementia. Such planning requires accurate projections of future numbers of people with dementia and future costs of providing quality care for them. This paper reports up-to-date projections to 2040 of numbers of older people with dementia and costs of their care in England.

A range of projections of future numbers of people with dementia and costs of care for dementia have been produced in recent years. The World Alzheimer’s Report 2015 [1] estimated that the number of people with dementia would rise from 47 million worldwide in 2015 to more than 131 million by 2050 and that total costs would cross the US$1 trillion threshold in 2018 and reach US$2 trillion by 2030. Brookmeyer *et al*. [2] projected that the prevalence of Alzheimer’s disease worldwide will quadruple to 101.5 million by 2050. Comas-Herrera *et al*. [3] projected that expenditure on long-term care services for older people with cognitive impairment in England will rise from 0.60% of Gross Domestic Product (GDP) (£5.4 billion) in 2002 to 0.96% of GDP (£16.7 billion) in 2031, under a set of specified assumptions. The Dementia UK report [4] estimated that the number of people with dementia in the UK would increase to over 1 million by 2025 and over 2 million by 2051 if age-specific prevalence remained stable.

The projections reported here are an advance on earlier projections in two main respects. First, they are based on detailed dynamic microsimulation modelling of future trends in risk factors and in cognitive function. Second, they are rooted in an analysis of the current costs of dementia care, which draws on detailed survey data on receipt of unpaid care and care services. They were produced as part of the MODEM (a comprehensive approach to MODelling outcome and costs impacts of interventions for DEMentia) study [5].

The context is the English National Health Service (NHS) and Adult Social Care (ASC). The NHS covers the entire population and funded through general taxation and is almost entirely free at point of use. Receipt of ASC services, however, is subject to both an assessment of care needs and a means test of incomes and savings.

## Data and methods

These cost projections are based on our estimate of the costs of care for older people in England in 2015 [6]. We estimate that the total cost was £23 billion in 2015 comprising £3.6 billion health care costs, £9.8 billion social care costs, of which £3.9 billion are met by local authorities and £5.9 billion by service users, and £9.5 billion opportunity costs of unpaid care by family and friends. The basis for this estimate and underlying data and methods are set out in our earlier paper [6].

The projections of future numbers of older people with dementia are based on Office for National Statistics (ONS) 2014-based principal population projections [7] and output of the the Population Ageing and Care Simulation (PACSim) dynamic microsimulation model [8], which simulates the characteristics (socio-demographic factors, health behaviours, chronic diseases and geriatric conditions) of individuals over the period of 2014–2040. Fuller details of PACSim and its validation are provided in the Appendix. PACSim produces projections of the prevalence of cognitive impairment by severity and level of need for care, separately by age group, gender and years of education.

The proportions of people with dementia receiving unpaid care and care services are estimated from our analyses of Cognitive Function and Ageing Study (CFASII) data and NHS Digital data. Data on the weekly costs of care are derived from our analyses of the MODEM cohort data [6]. Information on the CFASII and MODEM studies are provided in the Appendix.

Our projections model is a cell-based model in Excel with three parts. The first divides the older population by age (five age bands) and gender into subgroups (cells) by education (9 years or less, 10 or more years), household composition (three categories) and care setting (living in the community or a care home). To each of these cells is applied the proportion who have cognitive impairment projected by PACSim, to obtain an estimated number of older people with cognitive impairment. Data from CFASII on the proportion of people with cognitive impairment who have dementia is then applied to produce the estimated number with dementia by severity.

The second part produces projections of numbers of recipients of unpaid care and formal services. It divides the population into those receiving no care, unpaid care, formal community-based care, both unpaid and community-based care or residential care based on analysis of CFASII data. The proportions receiving care, by severity of dementia and other user characteristics, are held constant over time.

The third part produces projections of the opportunity costs of unpaid care, costs of health care and costs of social care (publicly or privately funded) for people with dementia. Data on weekly costs of care [6] are applied to projected numbers of service users. The resultant expenditure projections are then adjusted for expected real rises in the unit costs of care, such as the cost of a week’s residential care. Projected expenditure on social care is then divided between public expenditure by local authorities and private expenditure by service users using previous methods [9].

The MODEM study to which this paper relates received ethics approval from the Social Care Research Ethics Committee in London.

## Results

Our key base case assumptions are that the number of older will rise in line with official projections, prevalence of cognitive impairment and dementia will vary in line with PACSim projections, patterns of care will be unchanged and government policy will be unchanged. Our full set of assumptions is set out in Table 1.

**Table 1:** Base case assumptions

Demography
• The number of people by age and gender changes in line with the ONS 2014-based principal population projections [10]
• Marital status rates change in line with Government Actuary’s Department (GAD) 2008-based marital status and cohabitation projections to 2035 and then remain constant [14]
• There is a constant ratio of single people living alone to single people living with their children or with others
• The proportion of older people with 10 or more years of education rises in line with projections from the PACSIM model
Cognitive impairment/dementia
• Prevalence rates of cognitive impairment and of interval need for care by age, gender and education vary in line with projections from the PACSIM model [8]
• The proportion of cognitively impaired older people who have dementia remains constant by age group and gender
Care use
• The proportions of people with dementia receiving no care, unpaid care, formal community care services and residential care services remain constant for each sub-group by age, severity of dementia and other needs-related characteristics [6]
• The proportion of older service users with dementia whose care is privately funded remains constant [9]
Care costs
• The proportion of the costs of publicly funded care met by older service users through user charges remains constant
• Health and social care unit costs and the hourly opportunity cost of unpaid care rise in real terms in line with OBR [11] assumptions for future trends in productivity, with an uplift for the years to 2020 to take account of the planned rises in the national living wage (except that non-labour non-capital costs remain constant in real terms)
• Real Gross Domestic Product rises in line with OBR projections (2018)
• The supply of formal care will adjust to match demand and demand will be no more constrained by supply in the future than in the base year

### Numbers of older people with dementia

The ONS 2014-based principal population projections project that the overall older population of England aged 65 and over will rise from 9.7 million in 2015 to 11.7 million in 2025 and 15.3 million in 2040, a rise of 58% over 25 years [7]. The population aged 85 and over is projected to rise much more rapidly, by 138% over 25 years (Table 2).

**Table 2:** Projected number of older people with dementia receiving care and projected costs of dementia care in England, 2015–2040

	2015	2040	2015–40 (%)
Prevalence of dementia			
Number of people aged 65+	9,710,000	15,293,000	57%
Number of people aged 85+	1,297,000	3,083,000	138%
Number of older people with dementia	651,000	1,351,000	108%
Prevalence of dementia	6.7%	8.8%	32%
Mild dementia	110,000	167,000	52%
Moderate dementia	237,000	276,000	16%
Severe dementia	303,000	909,000	199%
Care recipients with dementia			
No care	120,000	170,000	42%
Unpaid care only	193,000	348,000	81%
Formal care only	21,000	37,000	71%
Both	65,000	131,000	100%
Care home residents	251,000	667,000	166%
Total annualised costs (£ million)			
Health care costs	3,530	10,310	192%
Social care costs	9,780	39,170	300%
Unpaid care	9,500	30,120	217%
Other costs	151	528	249%
Total	22,970	80,130	249%
Average costs (£ per person per year)			
Health care costs	5,440	7,630	40%
Social care costs	15,060	28,970	92%
Unpaid care	14,620	22,270	52%
Other costs	233	390	68%
Total	35,110	58,860	68%

Note: Numbers may not add exactly due to rounding.

The PACSim model projects that the prevalence rate of dementia in the older population will rise by almost one-third between 2015 and 2040 from 6.7% to 8.8%. We project that the numbers of older people with dementia will increase by 108%, from 650,000 in 2015 to 900,000 in 2025 and 1,350,000 in 2040 (Table 2). If rates of dementia by age and gender were to remain constant, the prevalence rate would be 7.2% in 2040 (rather than 8.8%) and the projected number of older people with dementia would be 1,100,000 in 2040, an increase of 70% between 2015 and 2040.

### Numbers of service users

The number of older people with dementia receiving no care, neither unpaid care nor formal services, is estimated to be around 120,000 in 2015 (Table 2). It is projected to rise to 125,000 in 2025 and 170,000 in 2040, an increase of 42% over 25 years. This is on the assumption of unchanged eligibility criteria for publicly funded care and unchanged probability of receiving unpaid care or purchasing care privately. The proportion of older people with dementia receiving no care is projected to fall from 18.4% in 2015 to 13.9% in 2040. This is because of the particularly large increase in the numbers with severe dementia: almost all those receiving no care have mild or moderate dementia.

We project the number of older people with dementia receiving unpaid care from family and friends to rise from 260,000 in 2015 to 331,000 in 2025 and 479,000 in 2040, an increase of 86% over the 25-year period (Table 2). The proportion of older people living in the community who receive unpaid care would remain broadly constant at two-thirds. In practice, the supply of unpaid care might not rise in line with demand [10], in which case the pressure on formal care services would be even greater.

We project the number of older people with dementia receiving community-based social services to rise from 86,000 in 2015 to 115,000 in 2025 and almost 168,000 in 2040, an increase of 95% over the 25-year period. These numbers imply 22% of older people with dementia living in the community receiving community-based services in 2015 rising to 27% in 2040, of whom around three-quarters receive unpaid care as well as formal care services.

Currently, around 251,000 older people with dementia live in care homes or hospitals, of whom 80% of them have severe dementia. We project the number in care homes to rise to 417,000 in 2025 and 667,000 in 2040, an increase of 166% over the 25-year period. The proportion of the older population with dementia living in care homes is projected to rise from 39% in 2015 to 49% in 2040, because of the projected rise in the numbers with severe dementia.

### Expenditure on care

**Figure 1:**
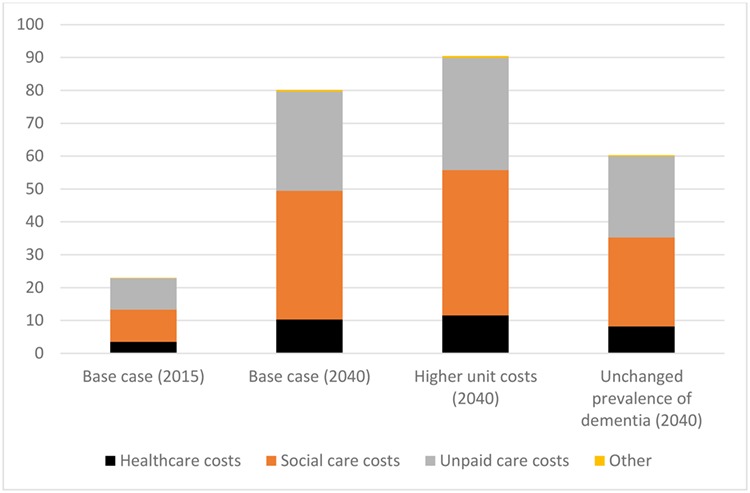
Projected health care, social care and unpaid care costs for older people with dementia: sensitivity analysis (£billion, at 2015 prices).

We project overall expenditure on care for older people with dementia to rise by 249% from around £23.0 billion in 2015 to £39.4 billion in 2025 and £80.1 billion in 2040 at constant 2015 prices (Table 2, Figure 1). When disaggregated, the total cost for healthcare is estimated to increase by 192% (from £3.5 billion in 2015 to £10.3 billion in 2040), social care by 300% (from £9.8 billion to £39.2 billion made up of £14.8 billion public and £24.4 billion private expenditure), and unpaid care by 217% (from £9.5 billion to £30.1 billion). Total costs for health care and social care for dementia are estimated to increase from 0.8% to 1.9% of GDP between 2015 and 2040. Unpaid care is not included in this estimate since it is not part of GDP.

If the prevalence rates of dementia remain constant (by age and gender) rather than vary in line with PACSim findings, projected expenditure would be £60.3 billion instead of £80.1 billion in 2040 (Figure 1).

These analyses assume that the unit costs of care, such as the cost of an hour’s home care, will rise from 2020 in line with Office for Budget Responsibility (OBR) assumptions for rises in average earnings [11] (somewhat faster to 2020 to account for the planned increase in the National Living Wage to 2020). There is scope for debate about whether wages in the care sector will rise in line with average earnings. If the real unit costs of care rose by 0.5% point per year faster than under the base case, expenditure is projected to be nearly 13% higher in 2040 than under the base case (£90.4 billion compared with £80.1 billion) (Figure 1).

We project that the average annual cost per person living with dementia will increase by 68%, from £35,110 in 2015 to £58,860 in 2040 (at 2015 prices), driven not only by the assumed real rise in the unit costs of care but also by an increase in the proportion of people with more severe dementia. The average annual cost per person with dementia in 2040 is projected to be £7630 for health care, £28,970 for social care and £22,270 for unpaid care (Table 2).

## Discussion

Our analyses project that the number of older people with dementia will more than double (108% increase) over the next 25 years and that overall expenditure on care for older people with dementia will more than treble (249% increase) at constant prices over the same period. An especially sharp rise, almost doubling, is estimated in the number of older people with severe dementia. As a result, the number living in care homes is projected to rise by 166% over the 25-year period, which in turn leads to a much faster increase in social care costs (92%) than in health care (40%) and unpaid care costs (52%) by 2040.

Our projections are an advance on those made in earlier studies. Instead of relying upon a priori assumptions about future trends in prevalence of dementia, our projections are based on detailed dynamic microsimulation of trends in cognitive function and draw on the most recent analyses of data from CFASII and the MODEM cohort. This is in line with a recommendation by Norton *et al*. [12] who called for increased investment in microsimulation to produce projections of future numbers of people with dementia. Our projections consider different levels of severity of dementia and are based on detailed analyses of the relationship between severity of dementia and receipt of unpaid care and care services [6].

Comparisons between our projections and those of previous studies need to be treated with some caution in view of differences in time periods, countries and health condition (Alzheimer’s disease, all dementias or cognitive impairment). Subject to this caveat, our projected increase in numbers of older people with dementia is broadly consistent with the World Alzheimer’s Report [1] and the Dementia UK report [4]. Our projected increase in expenditure is consistent with [5] but considerably greater than the World Alzheimer’s Report [1].

These findings need to be treated with some caution. They are not forecasts of the future, but projections based on a set of assumptions about future demographic, epidemiological and socio-economic trends. There is inevitable uncertainty about these trends. They are also based on current patterns of care and funding arrangements and do not assume any change in policy and practice. Moreover, no allowance has been made for changes in public expectations about the quality, range or level of care. In practice, public expectations and policies are likely to change.

Our projections assume that the supply of care will rise in line with projected demand. It is uncertain whether the supply of unpaid care will rise in line with demand, especially in view of higher rates of childlessness among future cohorts of older people. It is also uncertain whether increases in the unit costs of formal care broadly in line with rises in average earnings will be sufficient to ensure that the number of care staff rises in line with demand.

Despite these caveats, the overall message is clear that the costs of care for people with dementia, to health and social care budgets and to people with dementia themselves and their families, can be expected to rise rapidly in real terms over the next 25 years. This highlights the importance of measures to prevent or slow the progression of dementia including research to develop disease modifying treatments. It is also vital that we invest in research to develop clinically and cost-effective interventions to support people with dementia and their carers so that they can live well in their own homes and as independently as possible [13]. Action is needed now to gear up health and social care systems to cope with the future challenges.

## Supplementary Material

aa-19-0702-File003_afz154
