# Supplementary material for: Projections of care for older people with dementia in England: 2015 to 2040
Source: Age Ageing. 2019 Dec 6;49(2):264–9. doi: 10.1093/ageing/afz154 (PMC7047814; doi:10.1093/ageing/afz154)
Supplement: aa-19-0702-File003_afz154 [file aa-19-0702-file003_afz154.docx]

**Projections of Care for Older People with Dementia in England: 2015 to 2040**

**Appendix**

**PACSim methods**

PACSim is a discrete time dynamic microsimulation model that simulates the survival and characteristics (socio-demographic characteristics, health behaviours, chronic diseases and geriatric conditions, and care needs) of a set of real individuals (the base population) as they age over time, to estimate future prevalence, incidence, and life and health expectancies. The base population comprises three surveys: Understanding Society wave 1 individuals aged 35+ years); the English Longitudinal Study of Ageing (ELSA) wave 5 (individuals aged 50+ years); and the Cognitive Function and Ageing Study (CFAS) II (individuals aged 65+ years).

Apart from sex, education and socio-economic status, which were fixed, and age which was deterministic, all remaining characteristics were stochastic. Apart from dementia, transitions between states were determined by applying age, sex and state-specific transition probabilities derived from fitting binary, ordinal or generalised logistic regression models (dependent upon the characteristic) to the base and 2-year follow-up waves of the combined studies. The coefficients of each model were applied to current characteristics to produce the 2-year probability of moving to a given state; this was converted to a monthly probability for updating the characteristic in the simulation to achieve a more realistic evolution for characteristics that jointly influence each other.

Dementia status was available only in CFAS and was allocated probabilistically at the end of each year of simulation, from the results of a logistic regression model of dementia with age group, MMSE category and community/care home residence as covariates. Dementia did not contribute as an explanatory variable for the transition probabilities for other characteristics. Monthly survival probabilities were derived from the annual probabilities underlying the 2014-based principal population projection for England.

Validation of PACSim comprised comparison of: the numbers in 5-year age groups and life expectancy at age 65 at each year of the simulation with the Office for National Statistics 2014 projections for England; the age–sex-specific prevalence of stroke, diabetes, current smoking, overweight and obesity with those from the Health Survey for England 2014. Generally, there was good agreement apart from the prevalence of obesity where PACSim prevalence was lower by around 8 percentage points for men aged 35–64 and for women of all ages. Full details of PACSim methods and results of the validation have already been published^1,2^.

**References**

1. Kingston A, Robinson L, Booth H, Knapp M, Jagger C. Projections of multi-morbidity in the older population in England to 2035: estimates from the Population Ageing and Care Simulation (PACSim) model. Age Ageing. 2018;47(3):374-80.

2. Kingston A, Comas-Herrera A, Jagger C, Forecasting the care needs of the older population in England over the next 20 years: estimates from the Population Ageing and Care Simulation (PACSim) modelling study. Lancet Public Health 2018;3(9):E447-E455.

**The Cognitive Function and Ageing Studies (CFAS)**

The Cognitive Function and Ageing Studies (CFAS) are population based studies of individuals aged 65 and over living in the community or institutional settings. It comprises three main studies: MRC CFAS, the original study that began in 1989; the comparison study CFAS II (2008 onward) and CFAS Wales (2011).  The initial aims of CFAS were: to investigate dementia and cognitive decline in a representative sample of people aged over 65 years; to describe the service needs of people with dementia; to find out which factors increase the risk of someone developing dementia; and to investigate the different diseases that cause dementia and how quickly dementia progresses.

CFAS II, which is based in three centres in England (Cambridgeshire, Newcastle and Nottingham), builds on the design and infrastructure of CFAS I. Recruitment for CFAS II began in 2008. It provides baseline information on older people aged 65-84 in 2008-2011 who will reach the age of greatest frailty during the 2020s. CFASII data can be directly compared with the subset of MRC CFAS data known as CFAS I, which contains data collected at the identical three sites.

Further information on CFAS is available at <http://www.cfas.ac.uk/>

**A comprehensive approach to MODelling outcome and costs impacts of interventions for DEMentia (MODEM)**

The MODEM project explores how changes in arrangements for the future treatment and care of people living with dementia, and support for family and other unpaid carers, could result in better outcomes and more efficient use of resources. It funded by the Economic and Social Research Council (ESRC) and the National Institute for Health Research (NIHR) ^3^.

It aims to generate new evidence to inform policy and practice to better and more efficiently meet needs and promote health and wellbeing for people with dementia and their family and other carers. The project objectives include:

- Develop a suite of linked quantitative models, employing both microsimulation and macrosimulation techniques, to project future numbers of people with dementia, their dependency and other needs, comorbidities, levels of unpaid and formal care and associated expenditure;
- Gather evidence on the lives of people with dementia and their carers by collecting primary data from a new cohort, by conducting qualitative interviews and focus groups, and by examining data from previous trials and observational studies;
- Produce projections to 2040 of the numbers of older people with dementia in England, their needs for care and support, and associated public and private expenditure, together with projected outcomes and costs of a range of interventions to prevent or delay dementia incidence, slow symptom development, provide treatment and care, and support carers;
- Develop a Dementia Evidence Toolkit to make available evidence summaries of the effectiveness and cost-effectiveness of dementia care and treatment interventions and a searchable bibliographic database.

Further information on MODEM is available at <https://www.modem-dementia.org.uk/>

**Reference:**

3.Comas-Herrera A, Knapp M, Wittenberg R, Banerjee S, Bowling A, Grundy E, Jagger C, Farina N, Lombard D, Lorenz K, McDaid D on behalf of the MODEM Project group (2017) [MODEM: A comprehensive approach to modelling outcome and costs impacts of interventions for dementia. Protocol paper](https://bmchealthservres.biomedcentral.com/articles/10.1186/s12913-016-1945-x), *BMC Health Services Research*, 17, 1, 25
